# Decomposition of medical imaging spending growth between 2010 and 2021 in the US employer–insured population

**DOI:** 10.1093/haschl/qxae030

**Published:** 2024-03-27

**Authors:** Michal Horný, Daniel Chang, Eric W Christensen, Elizabeth Y Rula, Richard Duszak

**Affiliations:** Department of Radiology and Imaging Sciences, School of Medicine, Emory University, Atlanta, GA 30322, United States; Department of Health Policy and Management, Rollins School of Public Health, Emory University, Atlanta, GA 30322, United States; Department of Radiology and Imaging Sciences, School of Medicine, Emory University, Atlanta, GA 30322, United States; Harvey L. Neiman Health Policy Institute, Reston, VA 20191, United States; Health Services Management, University of Minnesota, St. Paul, MN 55108, United States; Harvey L. Neiman Health Policy Institute, Reston, VA 20191, United States; Department of Radiology, School of Medicine, University of Mississippi, Jackson, MS 39216, United States

**Keywords:** spending, imaging, employer-sponsored insurance, health care prices

## Abstract

Medical imaging, identified as a potential driver of unsustainable US health care spending growth, was subject to policies to reduce prices and use in low-value settings. Meanwhile, the Affordable Care Act increased access to preventive services—many involving imaging—for employer-sponsored insurance (ESI) beneficiaries. We used a large insurance claims database to examine imaging spending trends in the ESI population between 2010 and 2021—a period of considerable policy and benefits changes. Nominal spending on imaging increased 35.9% between 2010 and 2021, but as a share of total health care spending fell from 10.5% to 8.9%. The 22.5% growth of nominal imaging prices was below inflation, 24.3%, as measured by the Consumer Price Index. Other key contributors to imaging spending growth were increased use (7.4 percentage points [pp]), shifts toward advanced modalities (4.0 pp), and demographic changes (3.5 pp). Shifts in care settings and provider network participation resulted in 2.5-pp and 0.3-pp imaging spending decreases, respectively. In sum, imaging spending decreased as a share of all health care spending and relative to inflation, as intended by concurrent cost-containment policies.

## Introduction

US health care spending increased from $2.6 trillion in 2010 to $4.3 trillion in 2021 and is projected to grow, on average, by 5.4% per year over the next decade.^1-3^ This growth rate is considered by many to be unsustainable. Although policymakers have been mainly concerned about the solvency of public health insurance programs such as Medicare and Medicaid,^4^ spending growth in the employer-sponsored insurance (ESI) population is of similar importance. In particular, ESI spending directly translates to ESI premiums, the affordability of which for workers has to be supported by tax-exempt employer subsidies costing the federal government approximately $350 billion per year in lost federal tax revenue.^5,6^

Medical imaging has previously been asserted by some as a key contributor to health care spending growth.^7-9^ Although subsequent work documented that spending on medical imaging in traditional Medicare and ESI plans slowed considerably in the early 2010s,^10-13^ imaging has continued to be subject to various payment cuts^7,14-17^ and the target of measures to decrease use in low-value settings.^18^ Concurrently, the Affordable Care Act (ACA) increased access for ESI beneficiaries to high-value preventive services, many of which are imaging examinations; increased the size of the ESI population; and changed its demographic composition.

In light of these policy and health care delivery changes, we aimed to evaluate changes in spending on imaging relative to other medical services that occurred concurrently with changes in economic policy and insurance coverage to provide policymakers with more recent data on the drivers of medical spending in the ESI population, and the contribution of imaging to present trends.

## Data and methods

We used administrative records from the 2010–2021 Merative MarketScan Commercial Database (Merative, Ann Arbor, MI), which contain information on health plan enrollment as well as the use of and payments for covered inpatient and outpatient health care services and prescription drugs by ESI beneficiaries aged 0–64 years across the United States. We identified noninvasive diagnostic imaging services in the claims data using the Neiman Imaging Types of Services (NITOS), version 4.0 (Harvey L. Neiman Health Policy Institute).^19^ We also identified imaging-related services such as imaging contrast material and its administration using Healthcare Common Procedure Coding System codes A95*xx*, Q9951–Q9954, and Q9958–Q9967 billed on the same day as a contrast-enhanced imaging examination, and imaging-related facility fees as facility claims with a blank procedure code and a radiology-specific revenue code 0255, 0320–0329, 0350–0359, 0400–0409, 0610–0619, 0621, or 0972.

The data contained some missing values in the variables for patients’ location of residence and provider participation in patients’ health insurance network. In those cases, we approximated patients’ location of residence based on available geographic information in adjacent years and provider network participation based on whether the respective claim was paid by the patient's health plan using an in- or out-of-network payment rate. In the remaining cases, for which we could not approximate values based on available information, we replaced missing values using the hot-deck imputation technique.^20^ The frequencies of missing values are described in Table A1 in the online Supplementary material.

We measured nominal spending on medical imaging as the sum of payments from health plans and patients to health care providers for imaging and directly related services. To estimate population-level spending, we first calculated person-level metrics and subsequently applied methods for population inference from a complex survey design using statistical weights supplied by the MarketScan data provider. These annual statistical weights were constructed from the Public Use Microdata Sample of the American Community Survey to project annual MarketScan samples to the national populations of ESI beneficiaries.

To gain insight into factors that drove change in aggregate nominal spending on medical imaging between 2010 and 2021, we decomposed spending growth into the following: (1) change in nominal prices, (2) use of medical imaging per person, (3) imaging modality, (4) care delivery setting, (5) provider participation in patients’ health insurance network, and (6) patient demographics (changes in population size and relative distribution of age and sex within the population). We decomposed spending using methods used in prior research.^21^ For both 2010 and 2021, we calculated the number of beneficiaries for each combination of age and sex; the number of imaging services per person for each combination of patient age, sex, imaging modality, care delivery setting, and provider network affiliation; and an average price per service in each cell. Then, we calculated spending attributable to each factor as a difference between the aggregate spending recalculated using the 2021 value of the given factor while keeping all other factors at their 2010 levels and the actual aggregate spending in 2010.

We analyzed changes in nominal prices of imaging and non-imaging health care services over time using a data-driven market basket approach.^22^ To construct annual service baskets, we ranked health care services by spending and selected the top services that collectively accounted for 80% of the aggregate health care spending each year.^23^ Then, to estimate price growth, we fitted a generalized linear model with log link and gamma distribution functions and provider payment as the dependent variable to service-level data. The model included a triple interaction of a calendar year, a binary indicator of imaging vs non-imaging service, and a binary indicator of physician vs facility claim as the key independent variables of interest to capture possibly heterogenous annual price changes of imaging and non-imaging services billed by physicians and facilities. We adjusted the model using fixed effects for service billing codes including modifiers for professional and technical components, in-network vs out-of-network status of provider, whether the service was performed in an emergency department (ED) setting, whether the patient was younger than 18 years (because pediatric services tend to be more expensive than services provided to adults), and geographic area measured as the Metropolitan Statistical Area (MSA) or statewide non-MSA of the primary beneficiary's location of residence.

We counted imaging examinations as unique combinations of a patient, date of service, modality, and body region. This approach avoids double counting of examinations that were billed using professional and technical components instead of a global billing code and counts the number of services consistently over time despite the various coding changes that occurred during the study period (eg, the combination of previously separate billing codes for computed tomography [CT] of the abdomen and pelvis into single billing codes in 2011). Claims with procedure code modifier 59 (Distinct Procedural Service) were counted as separate examinations. Because the use of imaging is partially driven by the frequency of health care encounters, we also measured the use of imaging as a percentage of health care days (days when a patient used any health services) that involved imaging, percentage of health care users who received imaging, number of imaging examinations per imaging users, number of imaging days (days when a patient underwent imaging) per imaging user, and number of imaging examinations per imaging day.

Given the observational nature of this study and the use of de-identified data, this study was deemed oversight-exempt from the Emory University Institutional Review Board. The analysis was performed using SAS, version 9.4 (SAS Institute), and Stata, version 18.0 (StataCorp).

## Results

On average, there were 33.9 million covered lives annually in the sample from 2010 to 2021 (Table A2). Nominal spending for aggregate health care services increased 60.8% between 2010 and 2021. Nominal spending for medical imaging increased 35.9% while spending on all other health care services increased 63.7%. Accordingly, medical imaging's share of total health care spending fell from 10.47% in 2010 to 8.85% in 2021.

### Spending on medical imaging

**Figure 1.**
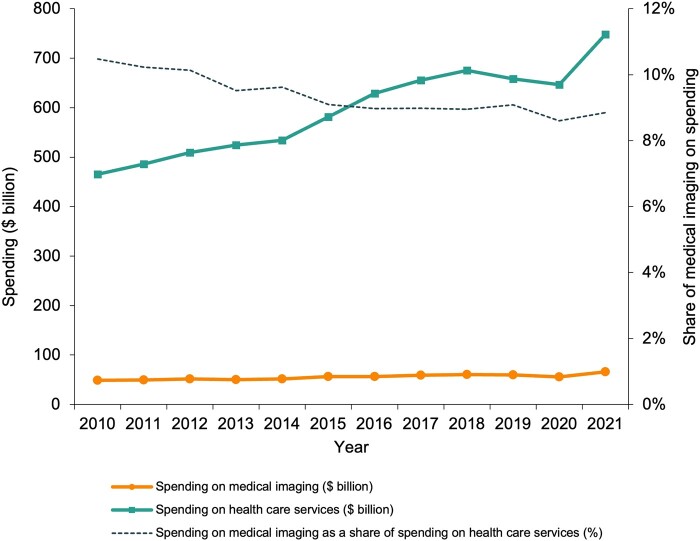
Nominal spending on medical imaging and all health care services in the ESI population from 2010 to 2021. The measure of spending on health care services excludes spending on prescription drugs. The 95% CIs for the displayed estimates are too narrow to be visible. They are reported in Table A3. Abbreviation: ESI, employer-sponsored insurance.

Nominal spending on medical imaging in the ESI population rose from $48.71 billion in 2010 (95% CI, $48.66 to $48.77 billion) to $66.19 billion in 2021 (95% CI, $66.06 to $66.33 billion) (Figure 1 and Table A3). The nominal spending on all inpatient and outpatient health care services grew from $465.10 billion (95% CI, $464.40 to $465.80 billion) to $748.15 billion (95% CI, $746.13 to $750.18 billion). Spending on medical imaging comprised 10.47% (95% CI, 10.46% to 10.49%) of spending on all health care services in 2010 and 8.85% in 2021 (95% CI, 8.83% to 8.87%). Among beneficiaries of plans with prescription drug coverage, imaging comprised 8.40% (95% CI, 8.39% to 8.41%) of spending on health care (inpatient and outpatient health care services and prescription drugs) in 2010 and 6.90% (95% CI, 6.89% to 6.92%) in 2021. Spending distribution across modalities, care delivery settings, and types of health care providers is reported in Tables A4–A6.

### Spending growth decomposition

**Figure 2.**
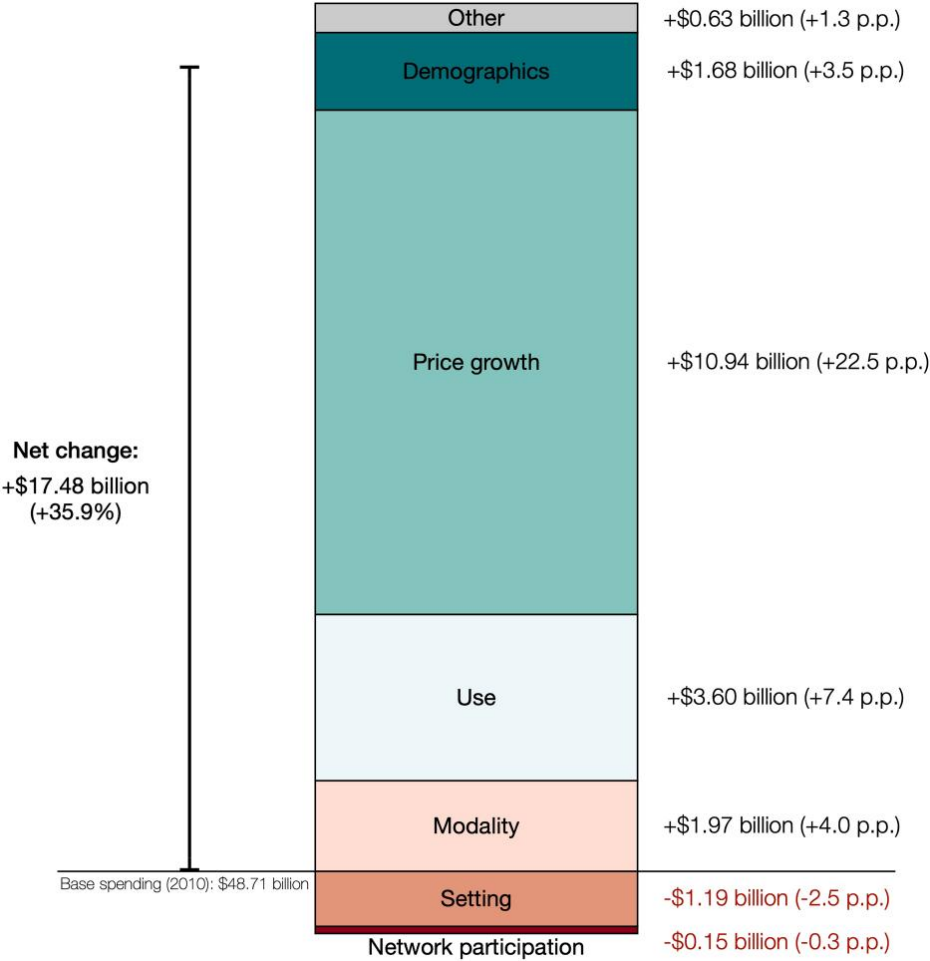
Decomposition of the growth of nominal spending on medical imaging in the ESI population between 2010 and 2021. The price inflation for medical imaging services between 2010 and 2021 was less than the general inflation of 24.26% measured by the Consumer Price Index.^24^ Abbreviations: ESI, employer-sponsored insurance; p.p., percentage points.

Between 2010 and 2021, nominal spending on medical imaging in the ESI population increased by $17.48 billion or 35.9% (Figure 2). This overall increase consisted of a $10.94 billion (22.5 percentage points [pp]) increase due to nominal price growth, a $3.60 billion (7.4 pp) increase due to higher use of medical imaging per capita, a $1.97 billion (4.0 pp) increase due to relative changes in use across imaging modalities, a $1.68 billion (3.5 pp) increase due to demographic changes in the ESI population, a $1.19 billion (−2.5 pp) decrease due to relative changes in settings where medical imaging was performed, a decrease of $154 million (−0.3 pp) due to a lower proportion of imaging services billed by out-of-network providers, and an additional $0.63 billion (1.3 pp) increase due to factors not accounted for by this analysis.

### Prices of medical imaging

**Figure 3.**
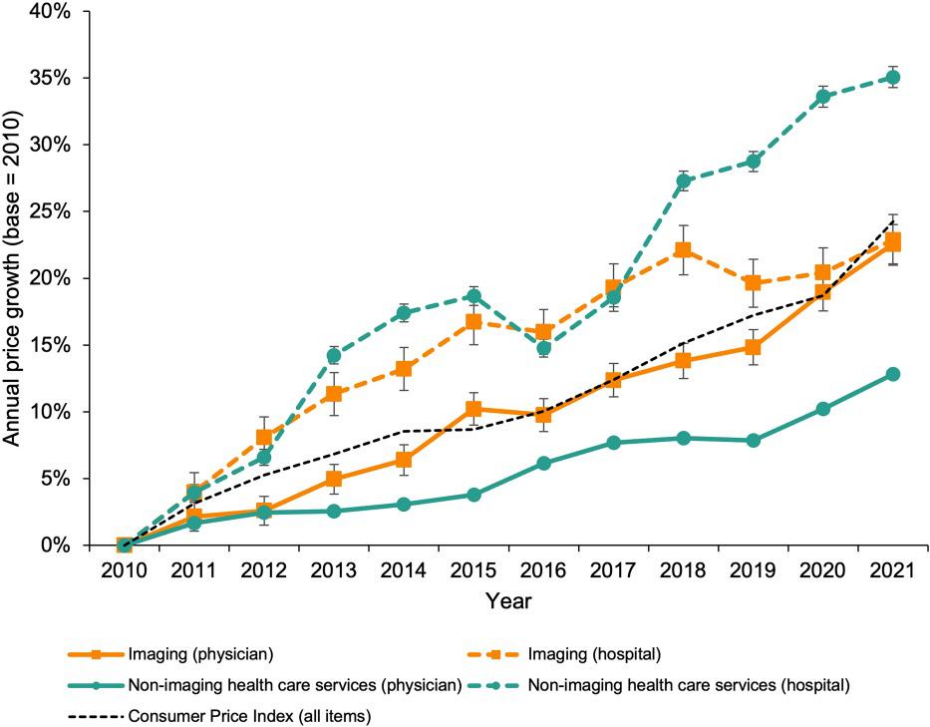
Price growth of imaging and non-imaging health care services in the ESI population from 2010 to 2021, by type of billing entity. Data on price inflation in the general economy measured by the Consumer Price Index were obtained from the US Bureau of Labor Statistics, Federal Reserve Bank of St. Louis.^24^ Abbreviation: ESI, employer-sponsored insurance.

From 2010 to 2021, nominal prices of hospital and physician imaging services increased by 22.87% (95% CI, 20.97% to 24.81%) and 22.56% (95% CI, 21.10% to 24.04%), respectively (Figure 3). Both growth rates were below the general inflation of 24.26% as measured by the Consumer Price Index.^24^ Prices of hospital and physician non-imaging services increased by 35.07% (95% CI, 34.27% to 35.88%) and 12.84% (95% CI, 12.52% to 13.16%), respectively. The growth of imaging prices was not uniform across modalities. While the nominal prices of nuclear medicine services increased by 39.90% (95% CI, 36.20% to 43.71%) between 2010 and 2021, the nominal prices of CT and magnetic resonance (MR) imaging increased only by 12.38% (95% CI, 11.30% to 13.46%) and 2.35% (95% CI, 1.27% to 3.44%), respectively (Figure A1).

### Use of medical imaging

The total number of imaging examinations performed in the ESI population increased from 143.56 million in 2010 (95% CI, 143.46 to 143.66 million) to 146.81 million in 2021 (95% CI, 146.63 to 146.99 million) (Table A7). While the proportion of ESI beneficiaries who use covered health care services increased from 75.35% in 2010 (95% CI, 75.34% to 75.37%) to 81.66% in 2021 (95% CI, 81.64% to 81.68%), the proportion of health care service users who use medical imaging services decreased from 46.16% (95% CI, 46.15% to 46.18%) to 40.31% (95% CI, 40.28% to 40.34%). Users of medical imaging services received, on average, 2.694 imaging examinations per year in 2010 (95% CI, 2.693 to 2.696) and 2.745 in 2021 (95% CI, 2.742 to 2.747). The proportion of health care days that involved imaging decreased from 12.84% (95% CI, 12.83% to 12.84%) to 9.80% (95% CI, 9.79% to 9.81%).

Although radiography/fluoroscopy remained the most frequent imaging modality, its relative share of overall medical imaging use in the ESI population decreased from 56.19% (95% CI, 56.16% to 56.21%) to 51.12% (95% CI, 51.08% to 51.16%) between 2010 and 2021 as did the relative share of nuclear medicine from 2.71% (95% CI, 2.70% to 2.71%) to 1.47% (95% CI, 1.46% to 1.48%) (Table A8). In contrast, the relative shares of ultrasound, CT, and MR imaging rose over this period: ultrasound from 24.01% (95% CI, 23.99% to 24.03%) to 27.94% (95% CI, 27.90% to 27.98%), CT from 10.17% (95% CI, 10.16% to 10.18%) to 12.09% (95% CI, 12.06% to 12.11%), and MR imaging from 6.92% (95% CI, 6.91% to 6.93%) to 7.38% (95% CI, 7.37% to 7.40%).

**Figure 4.**
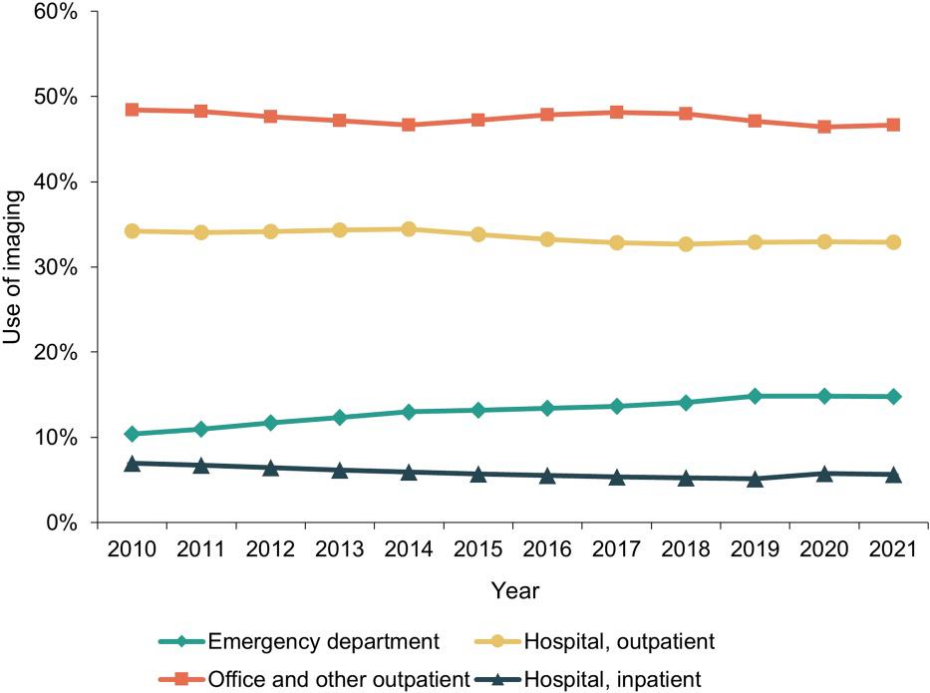
Relative distribution of the use of medical imaging across health care delivery settings in the ESI population from 2010 to 2021. The 95% CIs for the displayed estimates are too narrow to be visible. They are reported in Table A9. Abbreviation: ESI, employer-sponsored insurance.

The proportion of medical imaging examinations performed in ED settings increased from 10.37% (95% CI, 10.36% to 10.39%) in 2010 to 14.78% (95% CI, 14.75% to 14.81%) in 2021 (Figure 4 and Table A9). Simultaneously, the proportion of imaging examinations performed in other settings decreased over this period: hospital outpatient from 34.21% (95% CI, 34.19% to 34.24%) to 32.92% (95% CI, 32.88% to 32.96%), office and other outpatient settings from 48.45% (95% CI, 48.42% to 48.48%) to 46.67% (95% CI, 46.62% to 46.72%), and inpatient from 6.96% (95% CI, 6.94 to 6.99) to 5.63% (95% CI, 5.59 to 5.68).

The use of medical imaging temporarily decreased from 144.03 million examinations in 2019 (95% CI, 143.87 to 144.20 million) to 127.45 million in 2020 (95% CI, 127.29 to 127.61 million) due to the COVID-19 pandemic (Table A7). The drop was most pronounced in the office and other outpatient settings, where imaging use decreased by 12.7% from 67.83 million examinations in 2019 (95% CI, 67.74 to 67.92 million) to 59.20 million in 2020 (95% CI, 59.12 to 59.29 million) (Figure A2). As compared with 2019, the use of imaging in 2020 decreased by 11.8%, 11.3%, and 1.0% in the ED settings, hospital outpatient settings, and hospital inpatient settings, respectively.

### Demographic changes in the ESI population

Between 2010 and 2021, the ESI population size grew from approximately 153 million to over 162 million. The population size was relatively stable between 2010 and 2013 and grew mainly between 2014 and 2018. The most notable increases in the ESI population size were among young adults aged 21 to 26 years and individuals in their 30s (data not shown).

## Discussion

Over the 2010–2021 period, nominal spending for health care services increased 60.8% in the ESI population; however, spending growth in medical imaging was 35.9% compared to 63.7% for all other health care services. Hence, medical imaging's share of health care spending has declined from 10.5% to 8.9%. This finding demonstrates that medical imaging has not been a predominant driver of health care spending growth from 2010 to 2021. Decomposing the imaging spending growth, we found that growth in nominal prices was the largest contributor, accounting for nearly two-thirds of the spending growth, followed by increased utilization, shifts toward more advanced imaging modalities, and demographic changes.

Despite the fact that health care prices historically have grown faster than consumer prices in the general economy,^25^ inflation of imaging prices was on par with general price inflation. Notably, the difference in price growth between hospital and physician services was substantially less pronounced for imaging than for non-imaging services. These results align with existing evidence that hospital prices grow at a higher rate than physician prices,^26^ but demonstrate variability across types of services in both of these categories. Our analysis also showed that, among physician services, prices of imaging grew at a higher rate than non-imaging services. This may be explained by the increasing rate of physician practice acquisitions by large health systems in recent years on top of radiologists’ already frequent affiliation with hospitals.^27-29^ Although vertical integration in health care leads to increased prices,^29,30^ its impact on spending may be partially mitigated by the inherent alignment of hospital and physician participation in health insurance networks,^31,32^ or decreasing patients’ chances of encountering out-of-network physicians at in-network hospitals. We found that the proportion of imaging examinations that involved an out-of-network provider decreased from 12.1% in 2010 to 4.8% in 2021 and was associated with a decrease in spending by more than $150 million.

Our study also documented that price growth was slower for advanced imaging modalities, which are generally more expensive and thus have a disproportionate impact on spending. Specifically, nominal prices of CT grew more slowly than general inflation, and nominal prices of MR imaging stayed essentially constant since 2010. This finding suggests that ESI plans may have been deliberate when negotiating prices of health care services with providers, or potentially adopting similar targeted payment cuts as those imposed by the Centers for Medicare and Medicaid Services (CMS).^15-17,33^

The aggregate use of imaging was partially influenced by changes in the size and demographic composition of the ESI population, especially in the first years after implementation of the ACA. Various provisions of the ACA increased access to and take up of ESI,^34^ which increased the size of the ESI population.^35,36^ On the one hand, most of this population expansion occurred among young adults in their 20s and 30s, who are generally healthier and use fewer health care services than the rest of the population. On the other hand, the most common health-related condition in this age range is pregnancy, which often requires frequent use of imaging.^37^ Our model, which accounted for demographic shifts, estimated that these broad demographic changes led to a 3.5% increase in nominal spending on medical imaging between 2010 and 2021.

Some policies of the past decade aimed to increase the use of imaging in high-value settings, while others aimed to decrease use in low-value settings. Specifically, a provision of the ACA reduced financial barriers for ESI beneficiaries to access high-value preventive services, many of which are imaging examinations. Nonetheless, the uptake of imaging examinations for screening purposes did not increase meaningfully. For example, screening mammography rates in the ESI population did not change following the ACA,^38-40^ and the use of CT colonography for colorectal cancer screening increased only marginally following its recommendation by the US Preventive Services Task Force in 2016.^41^

In contrast, medical imaging was the subject of almost one-quarter of Choosing Wisely recommendations to reduce the use of unnecessary, low-value medical services.^18,42^ A systematic review demonstrated the intended effect was achieved for about two-thirds of recommendations aimed at reducing low-value imaging.^42^ Finally, the use of imaging per capita could also be influenced by technological advances, especially when approved by payers for reimbursement. For example, CMS and private payers adopted new billing codes for breast tomosynthesis in 2015, and development of novel tracers enabled more frequent use of positron emission tomography–CT in the management of patients with metastatic prostate cancer.^43^

While our findings cannot speak to the appropriateness of imaging use, our analysis revealed several noteworthy trends. The proportion of health care users who receive imaging declined from 46.2% to 40.3% over the study period. Similarly, the proportion of health care days that involved imaging declined from 12.8% to 9.8%. The less common utilization of imaging among patients actively seeking care suggests that patients’ presenting conditions warranted imaging less often, or health care providers were increasingly more judicious when deciding whether to order imaging for a patient at all. However, our analysis also showed that patients who underwent imaging received increasingly more imaging examinations. This growing imaging intensity should be carefully scrutinized to ensure a positive marginal value of each additional examination.

We also documented a trend of increasing use of imaging in ED settings, where care is expensive. The estimated net effect of changes in the relative distribution of care delivery setting on spending was negative primarily due to the decreasing use of imaging in the high-volume hospital outpatient setting, as well as because some of the increase in ED imaging use was already captured by the overall increase in imaging use.

Our findings extend and complement existing literature in various ways. This analysis provides novel evidence on recent trends in spending on medical imaging in the ESI population and identifies the key drivers of the observed trends. Previous analyses were based on data up to 2013, and thus could not capture the most recent demographic, market, policy, or care delivery changes.^13,44^ Similar to our results, 2 recent studies of Medicare Part B spending found that inflation-adjusted Medicare Part B fees for imaging declined, the number of imaging examinations slightly increased, and the share of imaging on overall spending decreased.^45,46^ Although those studies analyzed only outpatient procedures in a vastly different population, their findings coupled with ours suggest that changes in the use of and spending on imaging have been driven by broad changes in care delivery as they seem to be spanning across payers and populations.

### Limitations

This study was not without limitations. First, although annual MarketScan data captured 11%–36% of the entire ESI population throughout the study period, the samples were nonrandom and, as such, may have relayed biases from their composition. To minimize this potential bias, we applied statistical weights to demographic strata within our sample and thus ensured that the sample reflects the national ESI population on key observable characteristics. Second, because MarketScan contains only adjudicated claims, consumed health care services not billed through or covered by insurance were not captured. Consequently, our measures of use and spending may be underestimated. Third, a small, but nonnegligible, proportion of individuals in our sample were enrolled in fully or partially capitated health plans, some of which keep costs low—among other approaches—by reducing administrative burdens for providers and not requiring them to collect rigorous data on health care use. Although the collection of care use data by managed care plans coupled with fee-for-service equivalents has reportedly improved in recent years, our measures of use and spending may be underestimated.

Fourth, although most health care facilities in our sample disclosed how they distributed paid amounts to various revenue centers, allowing us to attribute spending to imaging vs non-imaging services, some facilities did not provide such a level of detail. In these cases, all payments for health care services to the facility were attributed to non-imaging services, and as such, our measures of spending on imaging may be slightly underestimated. Fifth, our data contained some missing values in certain key variables. When possible, we approximated these values using information available elsewhere, and replaced the remaining missing values using the hot-deck imputation technique. Sixth, we measured the use of imaging as a simple frequency of examinations, because it allows for straightforward interpretation. Nevertheless, treatment intensity is another important factor of the cost of care. Our analysis accounted for major changes in treatment intensity by analyzing utilization shifts across imaging modalities (eg, CT vs radiography/fluoroscopy) but may have missed smaller changes in treatment intensity within imaging modalities (eg, a shift from 2-dimensional mammography to 3-dimensional breast tomosynthesis). Finally, our observed changes in health care prices should be interpreted with caution, as some of this variation may be due to changes in the quality of care, which we were unable to measure.

## Conclusion

In conclusion, because medical imaging was previously identified as an important driver of health care spending growth, it was subject to various policies and initiatives aiming to reduce prices and discourage use in low-value settings.^7,14-18^ Although prior evidence suggested that some of these policies were more successful in achieving their goals than others,^16,42^ collectively they seem to have achieved the spending-reduction goal. Spending on medical imaging in the ESI population grew less than non-imaging services from 2010 to 2021. As such, policymakers working to curb health care spending should consider translating successful policies from imaging to other types of health care services, as long as patients maintain access to high-value care. As noted by others, targeting hospital prices may be the most effective way to curb spending growth, because hospital prices comprised the majority of spending growth. Nonetheless, opportunities to further decrease imaging spending may still exist. Specifically, the growth of imaging in the costly ED setting is concerning and deserves closer scrutiny, especially if some of these examinations could have been performed in more cost-effective non-emergency outpatient settings.

## Supplementary Material

qxae030_Supplementary_Data
