# Supplementary material for: Decomposition of medical imaging spending growth between 2010 and 2021 in the US employer–insured population
Source: Health Aff Sch. 2024 Mar 27;2(3):qxae030. doi: 10.1093/haschl/qxae030 (PMC10986240; doi:10.1093/haschl/qxae030)
Supplement: qxae030_Supplementary_Data [file qxae030_supplementary_data.zip › Imaging spending decomposition_v240227 - Appendix - clean.docx]

Appendix

Table A1: Frequency of missing values in key variables and the method of their replacement; by year

| Variable | Year | Missing values; N (%) |  |  |
| --- | --- | --- | --- | --- |
|  |  |  | Approximated by available information; N (%) | Replaced by hot-deck imputation; N (%) |
| Patients’ location of residence (enrollee level) | 2010 | 31  (0.0001%) | 31  (100.0%) | 0  (0.0%) |
|  | 2011 | 23  (0.00004%) | 23  (100.0%) | 0  (0.0%) |
|  | 2012 | 636  (0.001%) | 634  (99.7%) | 2  (0.3%) |
|  | 2013 | 193  (0.0004%) | 193  (100.0%) | 0  (0.0%) |
|  | 2014 | 692  (0.001%) | 690  (99.7%) | 2  (0.3%) |
|  | 2015 | 3,106  (0.02%) | 2,247  (72.3%) | 859  (27.7%) |
|  | 2016 | 15  (0.0001%) | 15  (100.0%) | 0  (0.0%) |
|  | 2017 | 2,539,419  (10.2%) | 2,127,517  (83.8%) | 411,902  (16.2%) |
|  | 2018 | 2,208,379  (8.4%) | 1,714,330  (77.6%) | 494,049  (22.4%) |
|  | 2019 | 1,675,086  (8.0%) | 1,158,558  (69.2%) | 516,528  (30.8%) |
|  | 2020 | 1,504,246  (7.8%) | 945,407  (62.8%) | 558,839  (37.2%) |
|  | 2021 | 3,176,247  (17.2%) | 2,130,971  (67.1%) | 1,045,276  (32.9%) |
| Provider participation in patients’ health plan network (service level) | 2010 | 119,265,847 (12.3%) | 36,921,846  (31.0%) | 82,344,001  (69.0%) |
|  | 2011 | 122,673,082  (11.6%) | 38,494,525  (31.4%) | 84,178,557  (68.6%) |
|  | 2012 | 123,879,016  (11.1%) | 39,933,752  (32.2%) | 83,945,264  (67.8%) |
|  | 2013 | 59,400,411  (6.9%) | 49,118,115  (82.7%) | 10,282,296  (17.3%) |
|  | 2014 | 104,691,776 (11.3%) | 98,392,318  (94.0%) | 6,299,458  (6.0%) |
|  | 2015 | 30,558,151  (7.2%) | 28,462,161  (93.1%) | 2,095,990  (6.9%) |
|  | 2016 | 31,243,167  (5.1%) | 28,463,051  (91.1%) | 2,780,116  (8.9%) |
|  | 2017 | 35,427,778  (6.6%) | 33,150,788  (93.6%) | 2,276,990  (6.4%) |
|  | 2018 | 39,352,456  (7.0%) | 37,323,566  (94.8%) | 2,028,890  (5.2%) |
|  | 2019 | 34,186,944  (7.6%) | 32,597,033  (95.3%) | 1,589,911  (4.7%) |
|  | 2020 | 29,738,985  (7.7%) | 27,345,446  (92.0%) | 2,393,539  (8%) |
|  | 2021 | 34,759,014  (8.0%) | 32,793,588  (94.3%) | 1,965,426  (5.7%) |

Table A2: Sample characteristics, by year

| Year | Sample size; N | Age;  Mean (SD) | Woman;  N (%) | Capitated or partially capitated health plan; N (%) | Health plan with prescription drug coverage; N (%) |
| --- | --- | --- | --- | --- | --- |
| 2010 | 50,837,688 | 32.96  (18.55) | 26,155,194  (51.4%) | 6,762,959  (13.3%) | 39,121,722  (77.0%) |
| 2011 | 53,881,348 | 33.01  (18.54) | 27,624,721  (51.3%) | 6,464,524  (12.0%) | 41,749,018  (77.5%) |
| 2012 | 55,260,701 | 33.06  (18.43) | 28,292,548  (51.2%) | 5,709,448  (10.3%) | 42,719,928  (77.3%) |
| 2013 | 42,974,913 | 33.14  (18.41) | 22,042,358  (51.3%) | 5,842,001  (13.6%) | 33,706,620  (78.4%) |
| 2014 | 46,313,829 | 33.29  (18.36) | 23,729,136  (51.2%) | 4,451,767  (9.6%) | 34,985,348  (75.5%) |
| 2015 | 19,553,498 | 33.11  (18.47) | 10,122,563  (51.8%) | 1,785,222  (9.1%) | 19,553,498  (100.0%) |
| 2016 | 28,614,815 | 33.20  (18.34) | 14,819,892  (51.8%) | 3,229,739  (11.3%) | 26,791,449  (93.6%) |
| 2017 | 24,849,846 | 32.84  (18.40) | 12,801,415  (51.5%) | 2,916,232  (11.7%) | 23,386,779  (94.1%) |
| 2018 | 26,158,337 | 32.93  (18.24) | 13,370,272  (51.1%) | 3,395,214  (13.0%) | 24,489,760  (93.6%) |
| 2019 | 21,062,097 | 32.85  (18.13) | 10,670,443  (50.7%) | 2,955,205  (14.0%) | 19,661,842  (93.4%) |
| 2020 | 19,342,676 | 32.94  (18.20) | 9,810,702  (50.7%) | 2,784,684  (14.4%) | 18,118,321  (93.7%) |
| 2021 | 18,506,785 | 32.93  (18.24) | 9,472,924  (51.2%) | 2,369,242  (12.8%) | 17,391,624  (94.0%) |

Table A3: Nominal spending on health care services and medical imaging in the ESI population from 2010 to 2021, by year

Notes: *) Among enrollees with prescription drug coverage.

| Year | Health care services; $ billion (95% CI) | Medical imaging; $ billion (95% CI) | Medical imaging as a share of health care services;  % (95% CI) | Medical imaging as a share of health care services and prescription drugs;*  % (95% CI) |
| --- | --- | --- | --- | --- |
| 2010 | 465.10  (464.40 to 465.80) | 48.71  (48.66 to 48.77) | 10.47  (10.46 to 10.49) | 8.40  (8.39 to 8.41) |
| 2011 | 485.67  (484.95 to 486.38) | 49.68  (49.62 to 49.73) | 10.23  (10.22 to 10.24) | 8.15  (8.14 to 8.17) |
| 2012 | 509.27  (508.54 to 510.00) | 51.59  (51.54 to 51.65) | 10.13  (10.12 to 10.14) | 8.12  (8.11 to 8.13) |
| 2013 | 524.54  (523.68 to 525.39) | 49.91  (49.85 to 49.97) | 9.51  (9.50 to 9.53) | 7.61  (7.60 to 7.62) |
| 2014 | 534.29  (533.39 to 535.20) | 51.42  (51.36 to 51.48) | 9.62  (9.61 to 9.64) | 7.49  (7.48 to 7.51) |
| 2015 | 618.22  (616.70 to 619.74) | 56.21  (56.11 to 56.31) | 9.09  (9.07 to 9.11) | 7.18  (7.17 to 7.20) |
| 2016 | 628.51  (627.12 to 629.91) | 56.40  (56.31 to 56.48) | 8.97  (8.96 to 8.99) | 7.11  (7.10 to 7.13) |
| 2017 | 655.18  (653.68 to 656.69) | 58.83  (58.73 to 58.93) | 8.98  (8.96 to 9.00) | 7.12  (7.10 to 7.13) |
| 2018 | 675.55  (674.02 to 677.09) | 60.48  (60.38 to 60.58) | 8.95  (8.93 to 8.97) | 7.09  (7.07 to 7.10) |
| 2019 | 658.25  (656.56 to 659.94) | 59.78  (59.67 to 59.89) | 9.08  (9.06 to 9.10) | 7.17  (7.15 to 7.18) |
| 2020 | 646.79  (644.89 to 648.68) | 55.63  (55.51 to 55.75) | 8.60  (8.58 to 8.62) | 6.63  (6.61 to 6.64) |
| 2021 | 748.15  (746.13 to 750.18) | 66.19  (66.06 to 66.33) | 8.85  (8.83 to 8.87) | 6.90  (6.89 to 6.92) |

Table A4: Relative distribution of the spending on medical imaging across imaging modalities in the ESI population from 2010 to 2021, by year

| Year | Computed tomography; % (95% CI) | Magnetic resonance; % (95% CI) | Nuclear medicine; % (95% CI) | Ultrasound; % (95% CI) | Radiography/ fluoroscopy; % (95% CI) |
| --- | --- | --- | --- | --- | --- |
| 2010 | 27.46  (27.42 to 27.50) | 22.44  (22.41 to 22.48) | 6.52  (6.50 to 6.54) | 20.50  (20.47 to 20.53) | 23.07  (23.05 to 23.10) |
| 2011 | 25.84  (25.80 to 25.88) | 22.88  (22.85 to 22.92) | 6.56  (6.53 to 6.58) | 21.56  (21.53 to 21.58) | 23.17  (23.14 to 23.19) |
| 2012 | 25.85  (25.81 to 25.89) | 22.79  (22.76 to 22.83) | 6.43  (6.41 to 6.45) | 21.94  (21.91 to 21.97) | 22.99  (22.96 to 23.02) |
| 2013 | 24.98  (24.94 to 25.02) | 22.62  (22.58 to 22.66) | 6.14  (6.11 to 6.16) | 23.08  (23.05 to 23.12) | 23.19  (23.16 to 23.22) |
| 2014 | 25.66  (25.61 to 25.70) | 22.06  (22.02 to 22.1) | 6.35  (6.32 to 6.37) | 23.08  (23.05 to 23.12) | 22.85  (22.82 to 22.88) |
| 2015 | 24.61  (24.55 to 24.67) | 22.46  (22.40 to 22.52) | 6.00  (5.97 to 6.04) | 24.17  (24.12 to 24.22) | 22.75  (22.71 to 22.79) |
| 2016 | 25.62  (25.56 to 25.67) | 21.88  (21.83 to 21.94) | 5.84  (5.81 to 5.87) | 24.61  (24.57 to 24.66) | 22.05  (22.01 to 22.08) |
| 2017 | 26.25  (26.19 to 26.31) | 21.30  (21.24 to 21.35) | 5.67  (5.64 to 5.70) | 24.68  (24.63 to 24.73) | 22.10  (22.07 to 22.14) |
| 2018 | 26.53  (26.47 to 26.58) | 20.62  (20.56 to 20.67) | 5.52  (5.49 to 5.56) | 24.88  (24.83 to 24.93) | 22.45  (22.41 to 22.49) |
| 2019 | 27.7  (27.63 to 27.77) | 19.57  (19.51 to 19.63) | 5.44  (5.41 to 5.48) | 24.41  (24.36 to 24.46) | 22.88  (22.83 to 22.92) |
| 2020 | 27.99  (27.91 to 28.07) | 19.79  (19.72 to 19.86) | 5.35  (5.31 to 5.39) | 24.48  (24.42 to 24.54) | 22.39  (22.34 to 22.44) |
| 2021 | 27.80  (27.73 to 27.87) | 19.74  (19.67 to 19.80) | 5.25  (5.21 to 5.29) | 24.43  (24.37 to 24.49) | 22.79  (22.74 to 22.84) |

Table A5: Relative distribution of the spending on medical imaging across health care delivery settings in the ESI population from 2010 to 2021, by year

| Year | Emergency department; % (95% CI) | Hospital, outpatient; % (95% CI) | Office and other outpatient;  % (95% CI) | Hospital, inpatient;  % (95% CI) |
| --- | --- | --- | --- | --- |
| 2010 | 15.49  (15.45 to 15.52) | 48.30  (48.26 to 48.35) | 29.41  (29.38 to 29.45) | 6.79  (6.75 to 6.83) |
| 2011 | 16.33  (16.30 to 16.37) | 48.58  (48.53 to 48.62) | 28.51  (28.48 to 28.55) | 6.57  (6.54 to 6.61) |
| 2012 | 17.62  (17.59 to 17.66) | 49.17  (49.12 to 49.21) | 26.76  (26.73 to 26.80) | 6.45  (6.41 to 6.49) |
| 2013 | 17.7  (17.66 to 17.74) | 48.39  (48.33 to 48.44) | 28.07  (28.03 to 28.11) | 5.85  (5.81 to 5.89) |
| 2014 | 19.41  (19.37 to 19.45) | 49.50  (49.45 to 49.55) | 25.5  (25.47 to 25.54) | 5.59  (5.55 to 5.63) |
| 2015 | 18.28  (18.22 to 18.34) | 48.77  (48.70 to 48.85) | 27.89  (27.83 to 27.95) | 5.05  (5.00 to 5.11) |
| 2016 | 20.01  (19.95 to 20.06) | 48.85  (48.78 to 48.92) | 26.21  (26.16 to 26.26) | 4.93  (4.88 to 4.98) |
| 2017 | 20.88  (20.82 to 20.94) | 48.21  (48.14 to 48.28) | 25.76  (25.71 to 25.81) | 5.16  (5.10 to 5.21) |
| 2018 | 21.75  (21.69 to 21.81) | 48.01  (47.94 to 48.08) | 25.29  (25.24 to 25.34) | 4.95  (4.9 to 5.00) |
| 2019 | 23.93  (23.86 to 24.00) | 47.10  (47.02 to 47.17) | 23.81  (23.76 to 23.86) | 5.16  (5.11 to 5.22) |
| 2020 | 23.26  (23.18 to 23.34) | 47.68  (47.58 to 47.77) | 23.85  (23.79 to 23.92) | 5.21  (5.15 to 5.28) |
| 2021 | 23.47  (23.40 to 23.55) | 47.75  (47.66 to 47.84) | 23.84  (23.79 to 23.90) | 4.93  (4.87 to 4.99) |

Table A6: Relative distribution of the spending on medical imaging across types of health care providers in the ESI population from 2010 to 2021, by year

| Year | Physician – Radiologist;  % (95% CI) | Physician – Non‑Radiologist;  % (95% CI) | Hospital;  % (95% CI) | Out-of-network providers;  % (95% CI) |
| --- | --- | --- | --- | --- |
| 2010 | 17.93  (17.91 to 17.95) | 22.47  (22.44 to 22.50) | 59.60  (59.57 to 59.64) | 10.47  (10.44 to 10.50) |
| 2011 | 17.15  (17.13 to 17.17) | 21.71  (21.68 to 21.73) | 61.14  (61.11 to 61.18) | 5.28  (5.25 to 5.31) |
| 2012 | 16.05  (16.03 to 16.07) | 20.83  (20.80 to 20.85) | 63.13  (63.09 to 63.16) | 4.79  (4.76 to 4.82) |
| 2013 | 18.19  (18.16 to 18.21) | 20.12  (20.09 to 20.15) | 61.70  (61.66 to 61.74) | 5.52  (5.49 to 5.55) |
| 2014 | 17.47  (17.45 to 17.49) | 18.15  (18.12 to 18.18) | 64.38  (64.34 to 64.42) | 4.62  (4.59 to 4.65) |
| 2015 | 17.74  (17.70 to 17.77) | 20.62  (20.57 to 20.67) | 61.64  (61.58 to 61.70) | 5.00  (4.96 to 5.05) |
| 2016 | 17.67  (17.64 to 17.70) | 18.97  (18.93 to 19.00) | 63.36  (63.31 to 63.41) | 4.71  (4.67 to 4.75) |
| 2017 | 17.08  (17.04 to 17.11) | 19.01  (18.97 to 19.05) | 63.91  (63.86 to 63.96) | 3.85  (3.81 to 3.88) |
| 2018 | 16.56  (16.53 to 16.59) | 19.54  (19.5 to 19.58) | 63.90  (63.85 to 63.96) | 4.04  (4.00 to 4.07) |
| 2019 | 16.38  (16.35 to 16.42) | 17.95  (17.91 to 17.99) | 65.67  (65.61 to 65.72) | 3.71  (3.68 to 3.75) |
| 2020 | 17.13  (17.09 to 17.17) | 18.01  (17.96 to 18.05) | 64.86  (64.80 to 64.93) | 3.70  (3.66 to 3.75) |
| 2021 | 17.53  (17.49 to 17.57) | 17.41  (17.37 to 17.45) | 65.06  (65.00 to 65.12) | 3.83  (3.79 to 3.87) |

Figure A1: Price growth of imaging services in the ESI population from 2010 to 2021, by modality

Notes: Data on price inflation in the general economy measured by the Consumer Price Index were obtained from the U.S. Bureau of Labor Statistics, Federal Reserve Bank of St. Louis.^24^

Table A7: Use of medical imaging in the ESI population from 2010 to 2021, by year

| Year | Imaging examinations; N million (95% CI) | Health care service users;  % (95% CI) | Imaging users as a share of health care service users;  % (95% CI) | Health care days with imaging; % (95% CI) | Imaging examinations per imaging user; Mean (95% CI) | Imaging days per imaging user;  Mean (95% CI) | Imaging examinations per imaging day; Mean (95% CI) |
| --- | --- | --- | --- | --- | --- | --- | --- |
| 2010 | 143.56  (143.46 to 143.66) | 75.35  (75.34 to 75.37) | 46.16  (46.15 to 46.18) | 12.84  (12.83 to 12.84) | 2.694  (2.693 to 2.696) | 2.341  (2.339 to 2.342) | 1.1510  (1.1508 to 1.1512) |
| 2011 | 144.72  (144.62 to 144.82) | 76.40  (76.38 to 76.41) | 45.89  (45.87 to 45.91) | 12.77  (12.76 to 12.77) | 2.689  (2.688 to 2.691) | 2.335  (2.334 to 2.336) | 1.1516  (1.1515 to 1.1518) |
| 2012 | 144.67  (144.57 to 144.76) | 77.24  (77.23 to 77.25) | 45.55  (45.54 to 45.57) | 12.54  (12.53 to 12.54) | 2.672  (2.670 to 2.673) | 2.323  (2.322 to 2.324) | 1.1502  (1.1500 to 1.1504) |
| 2013 | 141.91  (141.81 to 142.02) | 76.82  (76.8 to 76.83) | 44.99  (44.97 to 45.01) | 12.42  (12.41 to 12.42) | 2.675  (2.674 to 2.677) | 2.325  (2.323 to 2.326) | 1.1508  (1.1506 to 1.1510) |
| 2014 | 140.53  (140.42 to 140.63) | 76.34  (76.32 to 76.35) | 44.34  (44.32 to 44.36) | 12.31  (12.31 to 12.32) | 2.669  (2.667 to 2.670) | 2.314  (2.312 to 2.315) | 1.1535  (1.1533 to 1.1537) |
| 2015 | 148.93  (148.77 to 149.10) | 79.94  (79.92 to 79.96) | 44.30  (44.27 to 44.32) | 11.87  (11.86 to 11.88) | 2.674  (2.672 to 2.676) | 2.336  (2.334 to 2.337) | 1.1450  (1.1447 to 1.1453) |
| 2016 | 146.16  (146.02 to 146.29) | 78.83  (78.82 to 78.85) | 43.72  (43.70 to 43.74) | 11.79  (11.78 to 11.80) | 2.671  (2.669 to 2.673) | 2.332  (2.330 to 2.334) | 1.1453  (1.1451 to 1.1456) |
| 2017 | 147.72  (147.57 to 147.87) | 79.16  (79.14 to 79.17) | 43.29  (43.27 to 43.32) | 11.63  (11.62 to 11.64) | 2.676  (2.674 to 2.678) | 2.333  (2.331 to 2.335) | 1.1470  (1.1468 to 1.1473) |
| 2018 | 147.47  (147.33 to 147.62) | 78.79  (78.77 to 78.81) | 42.94  (42.92 to 42.97) | 11.53  (11.52 to 11.54) | 2.688  (2.686 to 2.690) | 2.339  (2.337 to 2.341) | 1.1493  (1.1490 to 1.1495) |
| 2019 | 144.03  (143.87 to 144.20) | 76.52  (76.50 to 76.54) | 43.01  (42.98 to 43.03) | 11.39  (11.38 to 11.40) | 2.687  (2.684 to 2.689) | 2.337  (2.335 to 2.339) | 1.1495  (1.1492 to 1.1498) |
| 2020 | 127.45  (127.29 to 127.61) | 76.53  (76.51 to 76.55) | 38.48  (38.45 to 38.51) | 10.18  (10.17 to 10.19) | 2.650  (2.647 to 2.653) | 2.300  (2.297 to 2.302) | 1.1525  (1.1521 to 1.1528) |
| 2021 | 146.81  (146.63 to 146.99) | 81.66  (81.64 to 81.68) | 40.31  (40.28 to 40.34) | 9.80  (9.79 to 9.81) | 2.745  (2.742 to 2.747) | 2.381  (2.378 to 2.383) | 1.1529  (1.1526 to 1.1532) |

Table A8: Relative distribution of the use of medical imaging across imaging modalities in the ESI population from 2010 to 2021, by year

| Year | Computed tomography; % (95% CI) | Magnetic resonance; % (95% CI) | Nuclear medicine; % (95% CI) | Ultrasound; % (95% CI) | Radiography/ fluoroscopy; % (95% CI) |
| --- | --- | --- | --- | --- | --- |
| 2010 | 10.17  (10.16 to 10.18) | 6.92  (6.91 to 6.93) | 2.71  (2.70 to 2.71) | 24.01  (23.99 to 24.03) | 56.19  (56.16 to 56.21) |
| 2011 | 10.14  (10.13 to 10.16) | 6.99  (6.99 to 7.00) | 2.51  (2.50 to 2.51) | 24.52  (24.50 to 24.54) | 55.83  (55.81 to 55.85) |
| 2012 | 10.15  (10.13 to 10.16) | 7.04  (7.03 to 7.05) | 2.34  (2.33 to 2.34) | 24.86  (24.84 to 24.88) | 55.62  (55.60 to 55.65) |
| 2013 | 9.94  (9.93 to 9.95) | 7.03  (7.02 to 7.04) | 2.15  (2.15 to 2.16) | 25.85  (25.82 to 25.87) | 55.03  (55.00 to 55.05) |
| 2014 | 10.28  (10.27 to 10.30) | 7.06  (7.05 to 7.07) | 2.10  (2.09 to 2.10) | 25.64  (25.62 to 25.66) | 54.92  (54.89 to 54.94) |
| 2015 | 10.17  (10.15 to 10.19) | 7.29  (7.27 to 7.30) | 1.96  (1.95 to 1.97) | 26.81  (26.78 to 26.85) | 53.76  (53.73 to 53.80) |
| 2016 | 10.60  (10.58 to 10.62) | 7.31  (7.30 to 7.33) | 1.84  (1.83 to 1.85) | 26.82  (26.79 to 26.85) | 53.43  (53.40 to 53.46) |
| 2017 | 10.88  (10.86 to 10.90) | 7.26  (7.24 to 7.27) | 1.76  (1.76 to 1.77) | 27.25  (27.21 to 27.28) | 52.85  (52.81 to 52.88) |
| 2018 | 11.19  (11.17 to 11.21) | 7.24  (7.23 to 7.25) | 1.70  (1.70 to 1.71) | 27.89  (27.86 to 27.93) | 51.97  (51.94 to 52.00) |
| 2019 | 11.49  (11.46 to 11.51) | 7.15  (7.13 to 7.16) | 1.65  (1.65 to 1.66) | 27.50  (27.46 to 27.54) | 52.21  (52.17 to 52.25) |
| 2020 | 11.93  (11.90 to 11.95) | 7.22  (7.21 to 7.24) | 1.54  (1.54 to 1.55) | 28.35  (28.31 to 28.40) | 50.95  (50.91 to 50.99) |
| 2021 | 12.09  (12.06 to 12.11) | 7.38  (7.37 to 7.40) | 1.47  (1.46 to 1.48) | 27.94  (27.90 to 27.98) | 51.12  (51.08 to 51.16) |

Figure A2: Use of medical imaging in the ESI population from 2010 to 2021, by health care delivery setting and year

Table A9: Relative distribution of the use of medical imaging across health care delivery settings in the ESI population from 2010 to 2021, by year

| Year | Emergency department; % (95% CI) | Hospital, outpatient; % (95% CI) | Office and other outpatient;  % (95% CI) | Hospital, inpatient;  % (95% CI) |
| --- | --- | --- | --- | --- |
| 2010 | 10.37  (10.36 to 10.39) | 34.21  (34.19 to 34.24) | 48.45  (48.42 to 48.48) | 6.96  (6.94 to 6.99) |
| 2011 | 10.96  (10.95 to 10.98) | 34.06  (34.04 to 34.08) | 48.25  (48.22 to 48.28) | 6.73  (6.70 to 6.75) |
| 2012 | 11.73  (11.71 to 11.74) | 34.19  (34.17 to 34.21) | 47.65  (47.62 to 47.67) | 6.44  (6.41 to 6.46) |
| 2013 | 11.99  (11.97 to 12.00) | 32.99  (32.97 to 33.02) | 48.89  (48.86 to 48.92) | 6.13  (6.10 to 6.16) |
| 2014 | 12.98  (12.97 to 13.00) | 34.43  (34.41 to 34.46) | 46.67  (46.64 to 46.70) | 5.92  (5.89 to 5.94) |
| 2015 | 11.99  (11.97 to 12.02) | 33.04  (33.00 to 33.07) | 49.40  (49.35 to 49.44) | 5.57  (5.53 to 5.61) |
| 2016 | 13.40  (13.38 to 13.42) | 33.23  (33.20 to 33.26) | 47.83  (47.79 to 47.87) | 5.54  (5.51 to 5.58) |
| 2017 | 13.65  (13.62 to 13.67) | 32.87  (32.83 to 32.90) | 48.12  (48.08 to 48.17) | 5.36  (5.33 to 5.40) |
| 2018 | 14.07  (14.04 to 14.09) | 32.68  (32.64 to 32.71) | 47.99  (47.95 to 48.03) | 5.27  (5.24 to 5.30) |
| 2019 | 14.87  (14.84 to 14.89) | 32.90  (32.86 to 32.94) | 47.09  (47.05 to 47.14) | 5.14  (5.10 to 5.18) |
| 2020 | 14.81  (14.78 to 14.85) | 32.98  (32.94 to 33.03) | 46.45  (46.40 to 46.50) | 5.75  (5.71 to 5.80) |
| 2021 | 14.78  (14.75 to 14.81) | 32.92  (32.88 to 32.96) | 46.67  (46.62 to 46.72) | 5.63  (5.59 to 5.68) |
